# Psychological Distress during Pregnancy in a Multi-Ethnic Community: Findings from the Born in Bradford Cohort Study

**DOI:** 10.1371/journal.pone.0060693

**Published:** 2013-04-30

**Authors:** Stephanie L. Prady, Kate E. Pickett, Tim Croudace, Lesley Fairley, Karen Bloor, Simon Gilbody, Kathleen E. Kiernan, John Wright

**Affiliations:** 1 Department of Health Sciences, University of York, York, United Kingdom; 2 Hull York Medical School, University of York, York, United Kingdom; 3 Bradford Institute for Health Research, Bradford Teaching Hospitals NHS Foundation Trust, Bradford, United Kingdom; 4 Department of Social Policy and Social Work, University of York, York, United Kingdom; Azienda Policlinico S. Orsola-Malpighi, Italy

## Abstract

**Purpose:**

Antenatal anxiety and depression are predictive of future mental distress, which has negative effects on children. Ethnic minority women are more likely to have a lower socio-economic status (SES) but it is unclear whether SES is an independent risk factor for mental health in pregnancy. We described the association between maternal mental distress and socio-demographic factors in a multi-ethnic cohort located in an economically deprived city in the UK.

**Methods:**

We defined eight distinct ethno-language groups (total N = 8,454) and classified a threshold of distress as the 75th centile of within-group GHQ-28 scores, which we used as the outcome for univariate and multivariate logistic regression for each ethnic group and for the sample overall.

**Results:**

Financial concerns were strongly and independently associated with worse mental health for six out of the eight ethnic groups, and for the cohort overall. In some groups, factors such as working status, education and family structure were associated with worse mental health, but for others these factors were of little importance.

**Conclusions:**

The diversity between and within ethnic groups in this sample underlines the need to take into consideration individual social, migration and economic circumstances and their potential effect on mental health in ethnically diverse areas.

## Introduction

Poor mental health during pregnancy can have serious ramifications for a mother and her family. Antenatal depression and anxiety can disrupt foetal developmental processes [1], [2] and are risk factors for postnatal maternal distress [3], [4] and children's poor functioning and their social, emotional and cognitive development [5]–[8].

There have been reports of increased risk of maternal antenatal, postnatal and general depression in some ethnic minority groups [9]–[12]. Ethnic minority women may face unique challenges that affect their psychological well-being, such as increased stress from discrimination. This may affect their mental health directly [13] or hinder their socio-economic status (SES) attainment [14], thus having an indirect detrimental effect on mental health [15]. However, some UK studies have illustrated that some groups of ethnic minority women have *better* mental health than the majority population [16], [17]. Potential explanations include strong family and community ties and effects of cohesion through increased ethnic density [18]–[20], or a ‘healthy migrant’ effect [17].

Ethnicity aside, lower SES is associated with postnatal depression [3], [4], but a recent review of 159 studies by Lancaster et al found mixed evidence for it being a risk factor for depressive symptoms during pregnancy [21]. While SES may moderate other risk factors antenatally, the lack of association could also be due to higher SES status and low variability in the antenatal populations studied [21]. Other risk factors for depressive symptoms in the Lancaster review included comorbid anxiety, previous depressive episodes, lack of social and partner support, pregnancy intent and not living with a partner. In addition to SES, inconsistent evidence was found for the effects of age and previous parity, which are likely to be correlated to education and income [22], obstetric history, substance use and, in this multi-country review, race/ethnicity [21]. There is some evidence that women who migrate to the UK may be at decreased risk for postnatal depressive symptoms, with increased length of residence associated with increased risk for symptoms [23]. Consanguinity is high in some South Asian cultures, and it may confer financial and social benefits such as improved family relationships [24].

In this study we report mental health during pregnancy in a large community birth cohort in Bradford, an ethnically diverse and economically deprived city in the North of England. We aimed to describe mental health for each ethnic group and the association between poor mental health and socio-demographic risk factors within those ethnic groups, and for the whole sample.

## Methods

### Population and recruitment

Born in Bradford (BiB) is a longitudinal multi-ethnic community birth cohort study aiming to examine the impact of environmental, psychological and genetic factors on maternal and child health and wellbeing [25]. Women were recruited while waiting for their glucose tolerance test, a routine procedure offered to all pregnant women registered at the Bradford Royal Infirmary, at 26–28 weeks gestation. For those consenting, the baseline questionnaire was collected via an interview held in a designated room with semi-private booths and conducted in English, Mirpuri (a spoken variant of Punjabi) or Urdu. Women not able to converse in any of these three languages did not complete the baseline questionnaire and are not included here. The full BiB cohort recruited 12,453 women during 13,776 pregnancies between 2007 and 2010 and the cohort is broadly characteristic of the city's maternal population [25]. Ethical approval for the data collection was granted by Bradford Research Ethics Committee (Ref 07/H1302/112).

### Translation

An initial Urdu translation of the baseline questionnaire was adapted for use as a script in this population by a professional translator through a three-version process of refinement using participatory methods [26], [27].

### Ethnicity and language of administration

Questions relating to ethnicity in BiB were based on those used in the UK's 2001 census and comprised one question asking which ethnic group the mothers considered themselves as belonging to (White, Mixed ethnic group, Black or Black British, Asian or Asian British, Chinese or other), followed by a further question, based on their response, about cultural background. The interviewer recorded the language in which the interview was conducted and we classified women according to ethnic group and language of administration.

### Mental health measurement

#### Administration and scoring

For the women completing the baseline questionnaire in English, the 28-item General Health Questionnaire (GHQ-28) [28] was administered as part of a self-completion module at the end of the interview. For those who chose to have the interview in Mirpuri or Urdu, the questions were read aloud and the interviewer coded the response. The GHQ-28 has a 4-item response scale anchored (typically) with ‘Not at all’, ‘No more than usual’, ‘Rather more than usual’, and ‘Much more than usual’ for negatively worded items, and ‘More so than usual’, ‘Same as usual’, ‘Rather less so than usual’ and ‘Much less than usual’, for the 8 positively worded items. We scored it using the GHQ method, (0-0-1-1) with higher scores indicating more distress (range 0 to 28). We allowed up to 4 missing GHQ-28 items, which we imputed with zero [29]. We considered more than 4 missing items to be indicative of the participant having a systematic problem with the GHQ-28 and excluded these few cases.

#### Determination of a threshold

Four related subscales have been identified in the GHQ-28; Somatic symptoms, Anxiety and Insomnia, Social Dysfunction and Severe Depression [28] however a psychometric analysis in this sample indicated that item response varied between ethnic groups and language of administration [30] so we did not use the subscale scores for this analysis.

The most commonly used threshold to detect psychiatric morbidity is ≥5 [31], but optimal thresholds between 3 and 8 have been reported [32], [33], with 6 or 7 for a small sample of English speaking BiB participants evaluated for depression (Mann et al, unpublished data). Due to uncertainty about the performance of the GHQ-28 in a non-English speaking and ethnic minority pregnant population we used a non-parametric determination of threshold [34] to indicate women at risk of worse mental health and set this at the 75^th^ centile score within each ethno-language group. As an overall indicator of risk factors across the population we also created a binary variable that classified as at risk all those determined to be at higher risk within their ethno-language group.

As a sensitivity analysis for determination of threshold scores we generated an internal indicator as proxy for a referent ‘gold standard’ using scores from the 14 items in the Anxiety and Insomnia (B) and Severe Depression (D) subscales. We attributed a score of zero to women who did not endorse any of the 14 items (90.5%) and a score of one to those who endorsed ≥4 items on the B subscale or ≥1 item on the D subscale (9.5%, ethnic group range 3.0% to 13.6%). We set the expected positive predictive value (PPV) for our sample at 45%, assuming a prevalence of 16% for any common mental disorder [32], [35]. We specified a bifactor model of the full dataset in MPlus version 5.21 to generate standardised general specific factor scores, and fitted loess smoothed curves against the B/D scoring threshold for each ethnic group, refitting these curves to the total GHQ-28 scores.

### Independent variables

All socio-demographic data were derived from the mother's baseline questionnaire except parity and gestational age. We classified age as those of average childbearing age (21–34 years), and those younger and older than this reference. We used parity (range 0 to 10) as gathered from the hospital maternity record, setting the reference category as zero, and other responses categorised as either 1–2 children or 3+. We used the mother's highest educational qualification and created a binary variable contrasting those with the equivalent of a maximum of 5 GSCE's (awarded at the end of compulsory education at age 16), unknown, or another qualification we could not classify, against those who achieved higher than 5 GCSE's. Some of our additional measures of SES were problematic in this sample as over 35% of the South Asian women did not know or did not report the amount of household income. Instead, we used the response to a question on financial security; “How well would you say you or you and your husband/partner are managing financially these days?” We categorised those ‘living comfortably’ or ‘doing alright’ against those ‘just about getting by’, ‘finding it quite difficult’ or ‘finding it very difficult’; classifying those who did not wish to answer (N = 31) as struggling financially. A second measure of financial security classified women as those behind or not behind with household bills, categorising ‘don't know’ or ‘don't wish to say’ separately. Working status we coded from four questions on employment status and classified as those working full time (reference category), working part time, not currently working, never worked and a full time student (regardless of working status). Those on maternity or sick leave were coded according to their working status, those missing a response on employment status but reporting the number of hours worked were assumed to be currently working. The relationship categories were married and living together, cohabiting (either status not necessarily with the baby's father), and not living with a partner. Consanguineous relationships were categorised as a positive response to the question about whether the mother was related to the baby's father other than by marriage. The modal number of people in the household varied considerably between ethnic groups and so we calculated tertiles within each group. Finally the country of birth and age of migration was classified as those who were UK born or moved when they were 16 or younger, and those who moved to the UK after age 16.

### Statistics

We tabulated socio-demographic status by ethnic group and then fitted univariate logistic regression models on the association between a covariate and being classified above the 75^th^ centile on the GHQ-28 score for each ethnic group. We then fitted a multivariate logistic regression model in each ethnic group, mutually adjusting for all covariates and also for gestational age at enrolment. Due to irrelevance or very low prevalence of some factors in some groups, we made some modifications to the classification of some covariates in some models (indicated in the results tables) Finally we fitted univariate and multivariate models for the whole sample, classifying all women above and below their within-ethnic group 75^th^ centile as binary outcomes in logistic regression models. We did not include consanguinity and age of migration as independent variables as they did not have relevance to some groups and recalculated the number of people in the household as tertiles for the whole sample. We present odds ratios (OR) with 95% confidence intervals (CI) and P-values, and set a threshold of alpha = 0.05 for statistical significance.

### Missing data

We used multiple imputation (MI) [36] to generate estimates to account for missing data in gestational age, parity and employment; these variables had >2% missing responses in one or more ethnic groups.

Because of potential differences in meaning and response by language of administration, we excluded those with no language of administration recorded (N = 35) and women from minority groups where there were too few (<100) cases to form an ethnicity group by language. To ensure independency of outcomes, we selected one questionnaire at random in cases where a woman had completed more than one GHQ-28 over the course of two or three enrolled pregnancies.

## Results

### Sample characteristics

**Figure 1:**
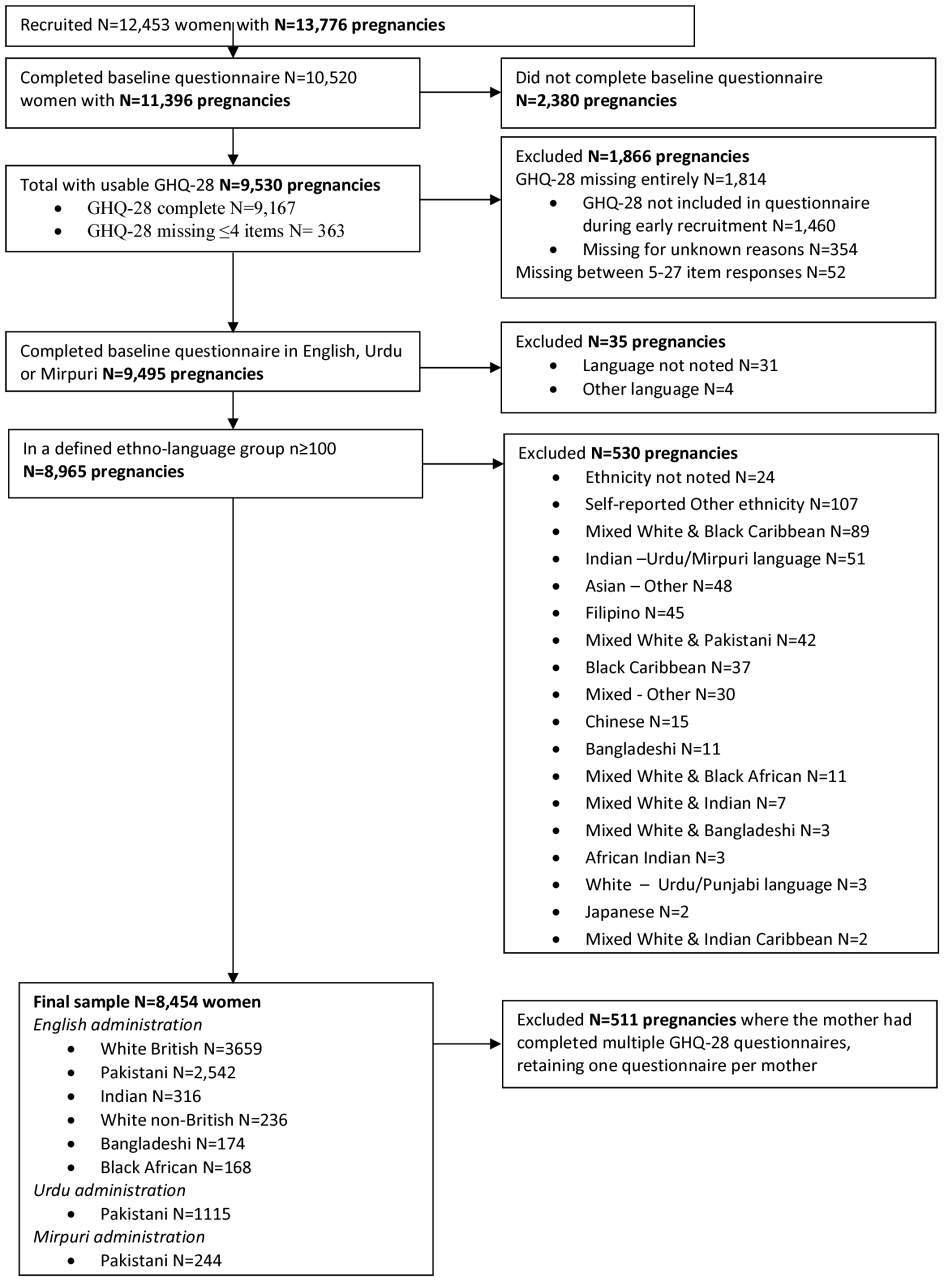
Study flowchart.

The derivation of the analysed sample is presented in Figure 1. Our achieved sample was 8,454 women; 80.4% of those with a baseline questionnaire. Of all the women with a questionnaire, there was little evidence that those included differed in age (t = 0.69, P = 0.49), or self-reported financial status (Chi^2^(5) = 6.1, P = 0.30) to those excluded.

### Ethnic and language groups

We defined eight distinct ethnic groups; six who completed the questionnaire in English and two Pakistani groups who completed the questionnaire in Urdu and Mirpuri.

### GHQ-28 scores

Within-group GHQ-28 75^th^ centile scores ranged from 7 for the Pakistani (Mirpuri language) and White non-British women to 11 in the Bangladeshi group (Table 1).

**Table 1:** Sample characteristics.

Language of questionnaire					English			Urdu	Mirpuri
Ethnicity		White British	Pakistani	Indian	White non-British	Bangladeshi	Black African	Pakistani	Pakistani
N (row %)		3659 (43.3)	2542 (30.1)	316 (3.7)	236 (2.8)	174 (2.1)	168 (2.0)	1115 (13.2)	244 (2.9)
*GHQ-28 (total score range 0–28)*	Median	4	6	3	3	5	4	5	4
	75^th^ centile	8	10	8	7	11	9	8	7
% with some imputed GHQ data		2.7	5.6	4.4	2.1	5.2	4.8	2.2	7.0
*Completed weeks of gestation, mean (SD)*		26.3 (1.9)	26.3 (2.0)	26.3 (1.5)	26.1 (1.8)	26.3 (2.1)	26.5 (2.1)	26.2 (2.1)	26.3 (2.6)
	missing*	3.0	2.9	4.8	6.4	1.7	13.1	2.2	1.6
*Age %*	Under 21	18.0	7.0	2.9	10.2	7.5	4.8	4.0	3.7
	21–34	66.7	79.6	81.7	81.4	79.3	80.4	81.0	73.8
	35+	15.1	13.2	15.2	8.5	13.2	14.9	14.8	22.5
	*missing*	0.2	0.1	0.3	0	0	0	0.2	0
*Employment status %*	Working full time	37.1	15.8	41.8	53.4	17.8	24.4	1.0	1.6
	Working part time	26.5	21.2	28.0	18.2	21.8	26.2	3.2	3.3
	Not currently working	24.5	33.9	16.5	13.1	26.8	17.3	8.7	6.2
	Never worked	8.0	25.5	12.3	7.6	19.0	18.5	85.6	80.3
	Full time student	3.9	2.8	2.9	7.2	3.5	13.1	1.1	2.5
	missing*	0.1	0.8	0.6	0.4	1.2	0.6	0.5	6.2
*Parity %*	Nulliparous	45.7	32.6	44.3	67.0	33.3	33.3	28.3	23.0
	1–2 other children	41.4	43.8	43.0	21.6	46.0	44.6	44.6	44.7
	3+ other children	6.9	17.0	5.4	3.0	16.1	4.8	21.0	28.7
	missing*	6.0	6.6	7.3	8.5	4.6	17.3	6.2	3.7
*Marital status %*	Married and living together	31.6	90.8	98.1	55.5	92.5	61.3	95.9	95.1
	Cohabiting	39.8	0.7	0.3	35.2	1.2	14.3	0.1	0
	Not living with a partner	28.5	8.4	1.6	9.3	6.3	24.4	3.9	4.9
	*missing*	0.1	0.2	0	0	0	0	0.2	0
*Related to baby*'*s father %*	No	99.8	39.8	90.2	99.6	78.7	98.2	32.4	27.9
	Yes	0.2	60.1	9.8	0.4	21.3	1.8	67.6	72.1
	*missing*	0	0.2	0	0	0	0	0	0
*Country of birth and age*	Migrated ≥16 years old	0.6	18.1	39.6	86.4	9.8	94.1	93.8	91.8
*at migration %*	Born in UK or migrated <16 years old	99.5	81.9	60.4	13.6	90.2	6.0	6.2	8.2
*Age migrants moved to UK, median (IQR)*		4 (2, 17)	17 (4, 21)	24 (20, 27)	23 (20, 25)	6 (3, 11)	24 (21, 28)	21 (19, 24)	21 (18, 24)
*Highest educational qualification, %*	<5 GCSE equivalent	20.0	15.2	5.7	5.5	16.7	9.5	39.2	50.8
	5 GCSE equivalent	34.0	30.3	16.1	15.3	32.8	20.8	31.2	26.6
	A-level equivalent	18.7	19.8	12.7	15.7	21.8	10.1	2.0	0.8
	Higher than A-level	18.7	29.4	56.3	45.3	24.7	43.5	26.1	13.9
	Other	7.4	4.3	7.9	15.7	2.9	14.9	0.9	0.4
	Unknown	1.2	0.9	1.0	2.5	1.2	0.6	0.4	7.0
	*missing*	0.03	0.1	0.3	0	0	0.6	0.4	0.4
*No. of people in household, median (IQR)*		3 (2 to 4)	4 (3 to 6)	3 (2 to 5)	2 (2 to 3)	4 (3 to 6)	3 (2 to 3)	5 (4 to 5)	6 (4 to 7)
*Financial wellbeing, %*	Not struggling financially	66.2	71.3	82.0	75.0	65.5	50.0	62.1	64.3
	Struggling financially	33.7	28.6	17.7	25.0	34.5	50.0	37.9	34.8
	*missing*	0.2	0.04	0.3	0	0	0	0	0.8
*Up to date with bills, %*	Yes	86.3	87.7	93.0	88.1	81.0	76.8	89.1	84.8
	No	11.8	8.8	4.8	10.6	14.4	21.4	6.5	7.0
	Don't know/doesn't wish to say	1.8	3.4	2.2	1.3	4.6	1.8	4.4	7.0
	*missing*	0.2	0.1	0	0	0	0	0.1	1.2

IQR interquartile range, SD standard deviation, GCSE General Certificate of Education (usually awarded at the end of compulsory education age 16), A-level Advanced Level (usually awarded at the end of two years of further education age 18),* missing data for these variables were imputed for regression models.

### Socio-demographic characteristics

There was a greater proportion of very young (<21 years) White British mothers (18%) compared to other groups (range 3–10%) and around a quarter of White British and Black African mothers were not living with a partner compared to fewer than 10% of the other groups. Around 65% of all Pakistani and 21% of Bangladeshi women were in a consanguineous relationship. The highest parity and largest households were found in the Urdu and Mirpuri groups. Over 90% of women in these two groups moved to the UK when they were 16 years of age and older, along with 94% of Black African and 86% of White non-British women, contrasting with only 10% of those of Bangladeshi origin. The Indian, White non-British and Black African groups appeared to have higher levels of educational attainment. Fewer Indian women reported struggling financially (18%) compared to the other groups (range White non-British 25% to Black African women 50%); similar trends were noted for those reporting being behind with bills.

### Within-group analyses

Univariate and multivariate analysis are presented in Tables 2 and 3 respectively.

**Table 2:** Risk factors for scoring over the within-group 75^th^ centile score, univariate analysis.

Language of questionnaire				English			Urdu	Mirpuri
Ethnicity	White British	Pakistani	Indian	White non-British	Bangladeshi	Black African	Pakistani	Pakistani
N^a^	3659	2542	316	236	174	168	1115	244
75th centile GHQ-28 score	8	10	8	7	11	9	8	7
Age 21–34	1	1	1	1	1	1	1	1
under 21	1.26 (1.04, 1.52)*	1.32 (0.95, 1.82)	2.76 (0.72, 10.6)	1.57 (0.65, 3.81)	0.82 (0.21, 3.14)	0.44 (0.05, 3.72)	0.64 (0.31, 1.30)	PP BMH(9)
35+	0.89 (0.72, 1.10)	1.21 (0.94, 1.54)	2.92 (1.54, 5.53)**	0.46 (0.13, 1.64)	0.96 (0.35, 2.63)	1.45 (0.58, 3.68)	1.00 (0.70, 1.43)	2.16 (1.14, 4.06)*
Working full time	1	1	1	1	1	1	0.84 (0.47, 1.51)	1.30 (0.47, 3.61)
Working part time	1.03 (0.85, 1.24)	1.12 (0.84, 1.50)	1.23 (0.65, 2.33)	0.80 (0.35, 1.85)	3.48 (1.00, 12.1)	1.60 (0.62, 4.12)	with above	with above
Not currently working	1.23 (1.02, 1.48)*	1.41 (1.08, 1.84)*	1.26 (0.61, 2.64)	1.59 (0.70, 3.67)	2.74 (0.85, 8.87)	1.00 (0.33, 3.03)	1	1
Never worked	1.39 (1.06, 1.83)*	1.02 (0.77, 1.35)	2.94 (1.39, 6.22)**	1.84 (0.66, 5.14)	1.75 (0.46, 6.66)	1.09 (0.37, 3.20)	1	1
Full time student	1.08 (0.73, 1.60)	1.83 (1.08, 3.09)*	0.43 (0.05, 3.57)	1.20 (0.39, 3.68)	3.24 (0.44, 23.8)	0.31 (0.06, 1.59)	1	1
Nulliparous	1	1	1	1	1	1	1	1
1–2 other children	0.92 (0.79, 1.08)	0.95 (0.78, 1.16)	1.13 (0.66, 1.93)	0.84 (0.39, 1.80)	1.29 (0.60, 2.77)	1.34 (0.60, 2.98)	1.52 (1.11, 2.08)*	2.18 (0.93, 5.08)
3+ other children	1.24 (0.93, 1.65)	1.08 (0.84, 1.38)	4.15 (1.49, 11.6)**	10.9 (1.44, 82.1)*	0.67(0.21, 2.07)	0.63 (0.08, 5.02)	1.75 (1.21, 2.52)**	3.40 (1.42, 8.10)**
Married & living together	1	1	1	1	1	1	1	1
Cohabiting	1.22 (1.02, 1.46)	1	1	1.59 (0.85, 2.97)	1	0.99 (0.35, 2.75)	1	1
Not living with a partner	1.60 (1.32, 1.93)**	2.08 (1.56, 2.76)***	1.83 (0.30, 11.2)	4.41 (1.73, 11.3)**	1.05 (0.27, 4.13)	0.96 (0.41, 2.21)	1.83 (0.99, 3.39)	2.74 (0.85, 8.82)
Not related to baby's father	N/A	1	1	N/A	1	N/A	1	1
Related to baby's father		0.81 (0.68, 0.96)*	1.13 (0.50, 2.66)		0.87 (0.37, 2.01)		0.87 (0.66, 1.14)	1.10 (0.59, 2.07)
Migrated ≥16 yrs. old	N/A	1	1	1	1	1	1	1
UK born/migrated <16 yrs. old		1.04 (0.83, 1.30)	1.12 (0.67, 1.86)	3.68 (1.71, 7.92)**	0.85 (0.28, 2.55)	0.74 (0.15, 3.62)	0.85 (0.49, 1.47)	2.29 (0.90, 5.80)
>GSCE equiv.	1	1	1	1	1	1	1	1
≤GSCE equiv./other/unknown	1.35 (1.61, 1.58)***	0.96 (0.81, 1.13)	1.43 (0.84, 2.41)	1.16 (0.65, 2.07)	0.58 (0.29, 1.14)	1.15 (0.57, 2.33)	1.08 (0.81, 1.44)	0.84 (0.39, 1.83)
Least no. of people in house	1	1	1	1	1	1	1	1
Middle tertile	1.04 (0.87, 1.24)	1.03 (0.84, 1.26)	1.17 (0.57, 2.37)	1.84 (0.92, 3.68)	1.24 (0.53, 2.91)	1.01 (0.44, 2.28)	1.15 (0.85, 1.56)	0.74 (0.38, 1.42)
Most no. of people in house	1.17 (0.98, 1.40)	0.91 (0.73, 1.14)	1.26 (0.72, 2.20)	1.23 (0.60, 2.52)	0.76 (0.34, 1.73)	1.69 (0.69, 4.11)	0.73 (0.53, 1.00)*	0.52 (0.25, 1.11)
Not struggling financially	1	1	1	1	1	1	**1**	1
Struggling financially	2.24 (1.93, 2.61)***	2.84 (2.37, 3.41)***	1.83 (0.99, 3.37)	3.09 (1.65, 5.77)***	2.17 (1.09, 4.34)*	2.18 (1.06, 4.49)*	2.07 (1.59, 2.68)***	2.85 (1.59, 5.10)***
Up to date with bills	1	**1**	1	1	1	1	1	1
Not up to date with bills	1.92 (1.55, 2.36)***	2.16 (1.63, 2.86)***	2.42 (0.85, 6.90)	2.36 (1.01, 5.51)*	2.02 (0.83, 4.90)	0.73 (0.29, 1.83)	2.71 (1.67, 4.39)***	2.79, 1.02, 7.62)*
Don't know/wish to say	1.86 (1.12, 3.08)*	1.55 (0.99, 2.41)	PP BMH(5)	6.00 (0.53, 67.5)	0.43 (0.05, 3.64)	PPWMH(3)	0.97 (0.51, 1.83)	2.20 (0.79, 6.08)

Estimates are OR (95% CI), P; *P<0.05, **P<0.01, ***P<0.001; bold indicates statistically significant estimates, N/A not applicable PP WMH (N) perfect prediction of worse mental health (N dropped from estimate), PP BMH (N) perfect prediction of better mental health (N dropped from estimate), N/A variable not analysed due to low applicability, ^a^N may vary slightly due to a small fraction of missing data.

**Table 3:** Risk factors for scoring over the within-group 75^th^ centile score, multivariate analysis.

Language of questionnaire				English			Urdu	Mirpuri
Ethnicity	White British	Pakistani	Indian	White non-British	Bangladeshi	Black African	Pakistani	Pakistani
N	3636	2525	313	236	174	166	1104	240
Age 21–34	1	1	1	1	1	1	1	1
under 21	1.14 (0.90, 1.45)	1.18 (0.80, 1.72)	1.58 (0.35, 7.08)	0.76 (0.23, 2.49)	0.57 (0.11, 3.01)	0.94 (0.08, 10.7)	0.96 (0.45, 2.04)	1
35+	0.97 (0.77, 1.22)	1.08 (0.81, 1.44)	2.69 (1.28, 5.67)**	0.21 (0.03, 1.28)	0.97 (0.31, 3.04)	1.08 (0.33, 3.57)	0.80 (0.53, 1.18)	1.33 (0.59, 3.02)
Working full time	1	1	1	1	1	1	0.93 (0.50, 1.70)	0.80 (0.23, 2.79)
Working part time	0.93 (0.75, 1.16)	1.16 (0.85, 1.58)	1.32 (0.65, 2.67)	0.77 (0.29, 2.03)	4.62 (1.19, 17.9)*	1.56 (0.54, 4.46)	with above	with above
Not currently working	0.81 (0.65, 1.03)	1.35 (1.00, 1.81)*	1.30 (0.57, 3.00)	0.59 (0.20, 1.76)	4.21 (1.17, 15.2)*	0.99 (0.29, 3.34)	1	1
Never worked	0.86, 0.63, 1.19)	1.01 (0.72, 1.40)	3.00 (1.21, 7.40)*	1.26 (0.38, 4.16)	1.89 (0.42, 8.42)	0.81 (0.24, 2.72)	1	1
Full time student	0.71 (0.46, 1.09)	1.67 (0.96, 2.89)	0.56, (0.07, 4.78)	0.64 (0.17, 2.43)	2.51 (0.26, 24.1)	0.29 (0.05, 1.69)	1	1
Nulliparous	1	1	1	1	1	1	1	1
1–2 other children	0.90 (0.71, 1.13)	1.03 (0.82, 1.30)	0.92 (0.49, 1.73)	0.65 (0.20, 2.12)	1.40 (0.54, 3.62)	1.21 (0.42, 3.50)	1.54 (1.09, 2.16)*	2.67 (0.97, 7.32)
3+ other children	1.10 (0.76, 1.60)	1.08 (0.77, 1.52)	2.09 (0.61, 7.12)	29.2 (1.67, 521)*	0.42 (0.08, 2.09)	0.46 (0.38, 5.58)	1.68 (1.07, 2.63)*	3.54 (1.14, 10.9)*
Married & living together	1	1	1	1	1	1	1	1
Cohabiting	1.07 (0.88, 1.29)	1	1	1.29 (0.62, 2.69)	1	0.99 (0.31, 3.07)	1	1
Not living with a partner	1.26 (1.01, 1.58)*	1.72 (1.26, 2.35)**	1.38 (1.78, 10.7)	3.22 (1.02, 10.1)*	1.17 (0.23, 5.96)	1.00 (0.04, 5.58)	1.58 (0.81, 3.08)	1.47 (0.38, 5.71)
Not related to baby's father	N/A	1	1	N/A	1	N/A	1	1
Related to baby's father		0.82 (0.67, 0.98)*	1.12 (0.45, 2.82)		0.79 (0.30, 2.07)		0.90 (0.67, 1.20)	1.31 (0.62, 2.74)
Migrated ≥16 yrs. old	N/A	1	1	1	1	1	1	1
UK born/migrated <16 yrs. old		0.89 (0.69, 1.14)	1.06 (0.60, 1.87)	1.84, (0.69, 4.91)	0.75 (0.20, 2.73)	0.62 (0.97, 3.99)	0.78 (0.44, 1.38)	2.58 (0.85, 7.84)
>GSCE equiv.	1	1	1	1	1	1	1	1
≤GSCE equiv./other/unknown	1.10 (0.92, 1.31)	0.81 (0.67, 0.99)*	0.94 (0.50, 1.77)	0.85, 0.42, 1.73)	0.62 (0.28, 1.36)	0.91 (0.42, 1.98)	1.05 (0.77, 1.43)	0.48 (0.20, 1.18)
Least no. of people in house	1	1	1	1	1	1	1	1
Middle tertile	1.10 (0.88, 1.40)	1.05 (0.82, 1.35)	0.96 (0.44, 2.11)	2.40 (0.84, 6.76)	2.55 (0.77, 8.42)	0.78 (0.27, 2.29)	1.17 (0.82, 1.67)	0.61 (0.28, 1.35)
Most no. of people in house	1.10 (0.88, 1.40)	1.06 (0.83, 1.35)	0.96 (0.51, 1.82)	0.98, 0.38, 2.52)	0.96 (0.40, 2.49)	1.57 (0.49, 5.07)	0.80 (0.57, 1.13)	0.51 (0.22, 1.17)
Not struggling financially	1	1	1	1	1	1	1	1
Struggling financially	2.04 (1.73, 2.40)***	2.65 (2.18, 3.22)***	1.30 (0.64, 2.65)	2.33 (1.10, 4.91)*	2.32 (1.04, 5.17)*	2.00 (0.81, 4.88)	1.81 (1.37, 2.38)***	2.14 (1.09, 4.18)*
Up to date with bills	1	1	1	1	1	1	1	1
Not up to date/Don't know/	1.47 (1.19, 1.82)***	1.50 (1.15, 1.95)**	0.90 (0.30, 2.69)	2.33 (0.92, 5.94)	1.66 (0.60, 4.63)	0.87 (0.33, 2.30)	1.42 (0.95, 2.14)	2.06 (0.90, 4.73)
doesn't wish to say								
Test of coefficients F(df), P^a^	**8.6 (16, 2.0^6^) <0.001**	**9.6 (12, 4.1^6^) <0.001**	1.3 (17, 4.0^6^) 0.18	1.6 (17, 2.7^5^) 0.07	1.1 (17, 3.2^6^) 0.40	0.6 (17, 3.7^5^) 0.91	**3.4 (14, 7.1^6^) <0.001**	**2.0 (13, 2.1^6^) 0.018**

Estimates are OR (95% CI), P after mutual adjustment and adjustment for gestational week, *P<0.05, **P<0.01, ***P<0.001; bold indicates statistically significant estimates, N/A not applicable, PP WMH (N) perfect prediction of worse mental health (N dropped from estimate), PP BMH (N) perfect prediction of better mental health (N dropped from estimate), df degrees of freedom (within-groups df inflated by multiple imputation), ^a^tests overall improvement in fit of the full model over the null (constant-only) model.

#### White British

Being less financially secure was independently associated with about a 2-fold risk of worse mental health. The following variables had univariate associations with worse mental health which were attenuated after full adjustment; <21 years old, not currently or ever working, not living with a partner and having less education.

#### Pakistani–English administration

Not currently working or being a student, not living with a partner and being less financially secure were associated with worse mental health, consanguinity was a protective factor. After adjustment all these effects persisted except working status, and less education emerged an independent protective factor.

#### Indian

Several variables showed univariate associations with worse mental health (being 35+, never having worked, having 3+ children and less education) however only being 35+ and never having worked showed independent associations with worse mental health after full adjustment. In the multivariate analysis only, there was a trebling of increased risk of worse mental health associated with never having worked compared to women working full time. However there was only weak evidence that the full model explained any variance over chance (P = 0.18).

#### White non-British

Several variables showed univariate associations with worse mental health; having 3+ children, not living with a partner, being UK born or migrating <16 years old and financial concerns. After adjustment, financial insecurity retained a significant association with worse mental health, there were some associations with parity and not living with a partner but very wide confidence intervals indicated instability within those estimates.

#### Bangladeshi

Although only struggling financially showed a univariate association with worse mental health, the fully fitted model indicated associations with working part time, not currently working and struggling financially. However there was little evidence that these covariates explained any variance over chance (P = 0.40).

#### Black African

Only struggling financially was associated with an increased risk of worse mental health on univariate analysis, in multivariate regression there was little evidence of association with any of the variables and adding the covariates did not improve the fit of the model (P = 0.91).

#### Pakistani–Urdu administration

Having more children and being financially insecure indicated strong and significant relationships with worse mental health, which persisted after full adjustment.

#### Pakistani–Mirpuri administration

Being older, having 3+ children, not living with a partner and being financially insecure had strong and significant relationships with worse mental health, of which only struggling financially and increased parity persisted after full adjustment.

### Overall model

We repeated the previous analysis using the classification of within-group at risk status as a binary outcome variable across groups. All covariates indicated an association with an increased risk of worse mental health, but only not being married, struggling financially, not being up to date with bills and having a larger household size persisted independently as risk factors after full adjustment (Table 4). With the exception of family size, these factors persisted after adjustment for ethnicity, indicating the general importance of these factors at a population level (Table 4). After adjustment for all other covariates, women who reported that they were struggling financially were 2.16 times more likely to be in the highest 25^th^ centile (95% CI 1.94, 2.40) than those who were not struggling financially.

**Table 4:** Univariate and multivariate regression of risk factors.

N = 8398	Univariate analysis	Multivariate analysis^b^	Multivariate analysis^c^
Age 21–34	1	1	1
Under 21	1.19 (1.03, 1.38)*	1.08 (0.91, 1.28)	1.11 (0.93, 1.32)
35+	1.08 (0.94, 1.23)	1.00 (0.87, 1.17)	1.02 (0.88, 1.19)
Working full time/part time	1	1	1
Not working/Full time student	1.22 (1.11, 1.45)***	1.02 (0.91, 1.14)	0.96 (0.85, 1.08)
First baby	1	1	1
1–2 other children	1.04 (0.93, 1.15)	1.02 (0.90, 1.15)	1.03 (0.91, 1.17)
3+ other children	1.33 (1.14, 1.54)***	1.15 (0.95, 1.39)	1.19 (0.98, 1.44)
Married & living together	1	1	1
Cohabiting	1.01 (0.89, 1.14)	1.01 (0.88, 1.17)	1.19 (1.01, 1.40)*
Not living with a partner	1.45 (1.28, 1.64)***	1.28 (1.11, 1.48)**	1.46 (1.24, 1.72)***
≤GSCE equiv./other/unknown	1	1	1
>GSCE equiv.	1.13 (1.03, 1.25)*	0.92 (0.82, 1.02)	0.93 (0.83, 1.03)
Least no. of people in house	1	1	1
Middle tertile	1.22 (1.07, 1.39)**	1.18 (1.03, 1.35)*	1.14 (1.00, 1.32)
Most no. of people in house	1.16 (1.04, 1.29)**	1.16 (1.01, 1.32)*	1.07 (0.93, 1.23)
Not struggling financially	1	1	1
Struggling financially	2.37 (2.14, 2.61)***	2.16 (1.94, 2.40)***	2.16 (1.95, 2.40)***
Up to date with bills	1	1	1
Not up to date with bills	1.98 (1.71, 2.29)***	1.45 (1.24, 1.70)***	1.48 (1.26, 1.73)***
Don't know/doesn't wish to say	1.51 (1.15, 1.98)**	1.28 (0.97, 1.69)	1.27 (0.96, 1.68)
Joint test of coefficients F, P^a^	-	24.2 (14, 4.1^6^) <0.0001	17.0 (21, 1.3^7^) <0.0001

Estimates are OR (95% CI), P; *P<0.05, **P<0.01, ***P<0.001, bold indicates statistically significant estimates, ^a^tests overall improvement in fit of the full model over the null (constant-only) model, ^b^Mutually adjusted and also adjusted for gestational age, ^c^as ^b^ and also adjusted for ethnicity.

### Sensitivity analysis for threshold

Plotting the internally generated ‘gold standard’ score against standardised factor scores revealed broadly similar slopes for all ethnic groups. At 45% PPV the range between the eight groups was 1.1 standard deviations (SD), with the range for the groups who answered English questionnaires at 0.6 SD. After refitting the curves using total GHQ-28 scores the indicated thresholds ranged between 13 and 17.5. The proportion scoring above these thresholds ranged from 14.5% (Pakistani women, English language administration), to 3.4% of the Pakistani women who completed the Urdu questionnaire and 2.0% of those completing it in Mirpuri. This very low prevalence in these latter two groups may reflect better mental health status in these more recent migrants, or a systematic difference to questionnaire response.

## Discussion

We have described the relationship between mental health and socio-economic and demographic characteristics in this multi-ethnic cohort of pregnant women living in an economically deprived UK city. Our study is unique in that it considers a large diverse community of women bounded by geography, which might imply greater commonality of risk factors than samples derived from population-wide estimates. We identified eight ethno-language groups, and for all groups except Indian and Black African women, struggling financially was strongly and independently associated with a 2-fold increased risk for worse mental health. In some groups, factors such as working status, education and family structure were associated with worse mental health, but for others these factors were of little importance. Our results highlight the complexity inherent in ascertaining individual, group and population risks in a multi-ethnic community.

### Categorisation of pathological distress

Prevalence estimates of depression and anxiety during pregnancy are usually considerably higher than estimates during the postnatal period. Consequently, calibration against diagnostic criteria often results in a lower threshold for optimal case-finding during pregnancy, e.g. [32], [37]. Possible explanations for these higher estimates include temporary worry over a pregnancy-related event and failure to attribute somatic symptoms to normal changes during pregnancy [38], [39]. This latter possibility is of particular interest in the GHQ-28 which includes many items related to sleep, fatigue, sub-optimal functioning or other somatic complaints.

Mindful of this, to improve specificity, we assumed that 25% of women in each group were at higher risk for worse mental health, but the error rate between our assumption and true caseness may have varied between ethnic groups. Data with which to compare these estimates are sparse, however a high burden of poor mental health in developing countries is generally indicated particularly among Pakistani [40], [41] and Bangladeshi women [42], and as our threshold scores were equal to or higher than those reported in diagnostic calibration studies [31]–[33] we considered we were favouring specificity over sensitivity.

We were unsure how cultural interpretation of the questions would influence responses [43] so we did not directly compare scores between ethno-language subgroups. Therefore we cannot report whether mental health advantage or disadvantage varies by ethnicity during pregnancy. Speculatively, there did appear to be some variation in 75^th^ centile scores, with highest scores among English-speaking Pakistani and Bangladeshi women. Our psychometric analysis indicated that the Pakistani women who completed the questionnaire in Mirpuri and Urdu had lower scores. If these high and low scores are indeed a true reflection of mental health, our data might concur with other research indicating worse mental health for migrant populations who have lived longest in the host country [17], [23]. However, data on the mental health of various ethnic minority groups living in the UK is far from congruent, with some surveys indicating a higher burden of distress among Indian [23], [44], Pakistani [9], [23], [44]–[46] and Asian women [12], others that South Asian women have less burden of illness [17], still others finding few differences in prevalence between Punjabi or White primary care attendees [47]. Explanations for differences include poor standardisation of screening and diagnostic instruments for cross-cultural use [17], [48], variation in classification of ethnic groups [49], and the consideration that the interaction between cultural, racial and ethnic identity might differ during the process of acculturation for individuals and populations [50].

### SES risk factors

While an association between poor mental health and low SES might be expected for general population samples e.g. [40], [51], our finding that financial concern is an independent risk factor is in contrast to the Lancaster review that examined risk factors for depression in pregnancy [21]. Several potential explanations are plausible.

Instead of absolute income we measured financial difficulty using both a subjective measure of financial concern and an indicator of household bill status. In support of this approach, results from another sample of low-income UK women with young children indicated that income-derived financial capability and financial difficulty measures had similar relationships with psychological distress [52]. Such non-income measures may have more direct bearing to the concerns, and thus the mental health of low income mothers [53] and recommend further methodological research in this area.

Our findings could be due to greater hardship and ethnic variation in this sample, as most studies in the Lancaster review were of relatively affluent women. If so, our results provide important evidence about a clear association between financial insecurity and poor mental health in a deprived, multi-ethnic population. However, we did not have details about mental health history, a factor shown in other studies to be a large, or largest, predictor of poor antenatal mental health [22], [54]. Relatedly, stress was not measured and social support variables were only available for a subsample; both have been associated with depressive symptoms in the maternal period in other studies [3], [55], [56]. Omitting such variables from our models may explain the persistence of the financial wellbeing component of SES in multivariate analysis.

For Indian, Bangladeshi and English-speaking Pakistani women, working status was independently associated with worse mental health. For the Indian women, the status of never having worked was associated with worse mental health but there were no strong associations for financial strain on mental health. Because our measure of employment was working status and not occupational class, we would have expected this to be related to, and explained by, the financial variables. However, our findings are supported with data from an analysis of a nationally representative dataset of UK ethnic minorities (EMPIRIC) collected in 2000 which found a burden of common mental disorders for an Indian-origin sample in the middle income group, not the lower income groups [57]. The Indian mothers in our sample were better educated and had lower levels of financial insecurity so other explanations such as social isolation may be important factors.

### Demographic risk factors

On bivariate analysis there was an association between not living with a partner and worse mental health for both White groups, English-speaking Pakistani women and for the sample overall. As only 8% of the English-speaking Pakistani women reported not living with a partner, the 2-fold univariate increase in risk represents a significant burden of mental distress in a small group that are more likely to be without crucial family support during their pregnancy. The persistence of this risk factor after full adjustment in these three groups and for the sample overall indicates ramifications of single parenthood over and above that of financial strain. The reason for the lack of association between the 25% of Black African women not living with their partner and worse mental health is unclear; for the Indian, Bangladeshi and Urdu and Mirpuri-speaking Pakistani groups the results may be limited by small numbers of non-partnered women.

For ethnic minority women, the association between being born in the UK and/or earlier age of migration and a higher risk for worse mental health have been confirmed in population datasets [17], and for mothers both 4–6 weeks [12] and 9-months postpartum [23]. We did not find strong associations with mental health and migration however migration patterns varied considerably. This highlights the need to consider both individual and ethnic group variation of the effect of migration patterns on mental health.

Black African women in this sample were recent migrants, reported high levels of financial concerns and non-cohabitation, however none of these factors, or any others we measured, were associated with worse mental health. Although this may be due to wide diversity of background and circumstances within the Black African categorisation [23], further research is clearly warranted in this population, particularly as asylum migration with an associated high distress burden is increasingly likely from African countries.

Consanguinity was common in the South Asian language groups and its emergence as a small but independent protective factor for Pakistani women who completed their questionnaire in English provides some evidence of potential benefits to mental health from closer familial ties in the diaspora. It is unclear, however, why no such effect was visible for the more recent migrants from Pakistan, or the Bangladeshi women.

Increased parity but not household size was independently associated with worse mental health for the Urdu and Mirpuri groups, although household size emerged as a risk factor for the overall sample which may indicate more commonalities across groups than we assumed.

### Strengths

Our study explores mental health in pregnancy, which is an important risk factor for negative consequences postnatally, in a sample of high ethnic diversity in a socio-economically deprived area. While the findings will not be generalizable to White, affluent communities, our results are likely to be relevant to many multi-ethnic urban settings. Being mindful to avoid category fallacy [17], we assumed a common prevalence of higher risk for mental distress across ethnic groups and not a common threshold score. Our sensitivity analysis indicated that this was a reasonable assumption for the English language groups.

### Limitations

Covariates did not improve fit over the empty model for several ethnic minority groups, indicating that explanatory risk factors may have been missing. This highlights the fact that the effect of structural situations and circumstances can vary considerably between ethnic minority groups. We lacked mental health diagnoses and our sensitivity analysis assumed similar prevalence of mental distress across groups, meaning we could not account for systematic differences in questionnaire response. The lack of a repeat mental distress measure may have inflated the number of non-distressed women we categorised as high risk [37], [38], [58], conversely the lack of association with covariates in some groups could have been caused by the threshold being set too high. Finally this cross-sectional analysis does not assess or imply causality.

### Conclusions

Our results highlight the importance of structural and social components such as poverty and family composition at the community level in a multi-ethnic pregnant population. The potential for diversity between and within ethnic groups in this community sample underscores the need to take into consideration individual social, migration and economic circumstances when planning mental health services in ethnically diverse areas. Further work is needed to further our understanding of the factors that contribute to mental health, and distress, at a variety of community and population levels.
